# A Multi-Omics Analysis of Mucosal-Associated-Invariant T Cells Reveals Key Drivers of Distinct Modes of Activation

**DOI:** 10.3389/fimmu.2021.616967

**Published:** 2021-05-24

**Authors:** Kristin Schubert, Isabel Karkossa, Jana Schor, Beatrice Engelmann, Lisa Maria Steinheuer, Tony Bruns, Ulrike Rolle-Kampczyk, Jörg Hackermüller, Martin von Bergen

**Affiliations:** ^1^ Department of Molecular Systems Biology, Helmholtz-Centre for Environmental Research (UFZ), Leipzig, Germany; ^2^ Department of Medicine III, University Hospital Rheinisch-Westfälische Technische Hochschule Aachen (RWTH), Aachen, Germany; ^3^ Institute of Biochemistry, Leipzig University, Leipzig, Germany

**Keywords:** MAIT cells, multi-omics analysis, TCR-dependent, TCR-independent, activation, key driver analysis

## Abstract

The function of mucosal-associated invariant T (MAIT) cells highly depends on the mode of activation, either by recognition of bacterial metabolites *via* their T cell receptor (TCR) or in a TCR-independent manner *via* cytokines. The underlying molecular mechanisms are not entirely understood. To define the activation of MAIT cells on the molecular level, we applied a multi-omics approach with untargeted transcriptomics, proteomics and metabolomics. Transcriptomic analysis of *E. coli*- and TCR-activated MAIT cells showed a distinct transcriptional reprogramming, including altered pathways, transcription factors and effector molecules. We validated the consequences of this reprogramming on the phenotype by proteomics and metabolomics. Thus, and to distinguish between TCR-dependent and -independent activation, MAIT cells were stimulated with IL12/IL18, anti-CD3/CD28 or both. Only a combination of both led to full activation of MAIT cells, comparable to activation by *E. coli*. Using an integrated network-based approach, we identified key drivers of the distinct modes of activation, including cytokines and transcription factors, as well as negative feedback regulators like TWIST1 or LAG3. Taken together, we present novel insights into the biological function of MAIT cells, which may represent a basis for therapeutic approaches to target MAIT cells in pathological conditions.

## Introduction

In the last decade, innate-like T cells (ITC) gained emerging recognition as important players in the early immune response to infections. ITCs constitute 10-20% of human T cells, with mucosal-associated invariant T (MAIT) cells being one of the most abundant ITCs in humans (1). MAIT cells have been linked to the pathogenesis of several diseases, e.g., inflammatory bowel diseases (2), type 1 diabetes (3), rheumatoid arthritis (4) or inflammatory liver diseases (5, 6), where they acquire an activated phenotype. Importantly, drugs and drug-like molecules can modulate MAIT cell activation (7), rendering them attractive targets for therapeutic approaches, requiring a deep and detailed understanding of mechanisms involved in the activation of MAIT cells.

Under healthy conditions, MAIT cells accumulate at mucosal barrier sites, e.g., in the gut lamina propria (8) or the liver (9) and are considered to have a critical immune surveillance function, which requires them to distinguish between commensal and pathogenic bacteria. During infection, MAIT cells have been shown to respond rapidly, even without prior antigen exposure through antigen-T cell receptor(TCR)-dependent and -independent mechanisms, mediated mainly by the innate cytokines IL-12 and IL-18 (10). Through their semi-invariant TCR Vα7.2-Jα33 (8), MAIT cells recognize small molecules like metabolites from the bacterial riboflavin pathway, which are presented by the MHCI-like molecule MR1 (11). A robust activation, however, occurs only by a combination of both TCR-dependent and -independent mechanism (12, 13) that *in vitro* can be achieved by activating MAIT cells with, e.g., *E. coli*-pulsed antigen-presenting cells (14).

Activation of MAIT cells is accompanied by the release of cytotoxic effector molecules like granzyme A, B, K or perforin, and the proinflammatory cytokines IFN-γ, TNF and IL-17 (15). Release of several additional inflammatory mediators has been postulated (4, 16), but molecular mechanisms and underlying pathway alterations during MAIT cell activation are only at the beginning of understanding. With the use of high-resolution omics data, deep profiling of immune cells has added a global view on molecular mechanisms underlying immune cell biology and function (17, 18), giving novel insights that can be utilized for therapeutic approaches. For MAIT cells, transcriptomic (1, 5) and proteomic (16) strategies have been applied to some extent, already defining the effector function in the resting state (1, 5, 16) on the molecular level. Recently, transcriptional profiling has revealed that MAIT cell function highly depend on the mode of activation (19). While TCR-dependent activation results in a quick and strong response, TCR-independent activation showed a delayed and limited response. However, molecular profiling of a combination of both stimuli that are also present under inflammatory conditions is lacking.

Here, we present a multi-omics approach using global transcriptomic, proteomic and metabolomic profiling to define MAIT cell activation. We used an *ex vivo* model of MAIT cell activation to investigate TCR-dependent and -independent activation and analyzed pathway alterations, as well as the transcription factor and effector molecule profile. Moreover, by using an integrated network-based analysis, we identified key drivers of MAIT cell activation.

## Materials and Methods

### Cell Culture

Healthy adult blood was obtained from the blood bank of the University Leipzig, approved by the local ethics committee (Ref. #079-15-09032015). Peripheral blood mononuclear cells were isolated from male human donors at age 20-50 by Ficoll-Paque™ density-gradient (GE Healthcare, UK) centrifugation. CD161+ TCR Vα7.2+ MAIT cells and CD14+ monocytes were obtained by positive magnetic separation using respective microbeads (Miltenyi Biotec, Germany). For RNA-Seq experiments, monocytes were loaded with fixed *E. coli* at a MOI of 10 for 3h, followed by intense washing in PBS to remove *E. coli*. MAIT cells and *E. coli*-loaded monocytes were incubated at a ratio of 1:1 for 16h. In some experiments, MAIT cells were stimulated with 50ng/ml IL-12 and 50ng/ml IL-18 (both from MBL International, MA, USA), 10µg/ml plate-bound anti-CD3 and 1µg/ml soluble anti-CD28 (both from Biolegend, CA, USA) or a combination of both for 16h. For TWIST1 inhibition experiments, the small molecule inhibitor harmine (Sigma-Aldrich, Germany) was used at a concentration of 20 or 40µM, DMSO served as solvent control.

### Preparation of Bacteria

The *E. coli* stain K12 MG1655 was purchased from DSMZ, Germany. Bacteria were grown to stationary phase in LB medium at 37°C. Bacteria were washed in PBS, fixed in 1% formaldehyde for 5 min, followed by 3 washes in PBS. Fixed bacteria were resuspended in RPMI for further use.

### Flow Cytometry and Cell Sorting

For immunophenotyping, cells were stained in PBS supplemented with 0.5% BSA and 2mM EDTA for 15min at 4°C with following fluorochrome-conjugated antibodies: anti-CD69-FITC or anti-CD3-FITC, anti-TCR Vα7.2-PE, anti-CD161-APC (all from Miltenyi Biotec, Germany), anti-CD223(LAG3)-FITC, anti-CD154(CD40L)-FITC (Biolegend, USA). Dead cells were excluded using propidium iodide (Miltenyi Biotec, Germany). MAIT cells were identified based on their TCR Vα7.2 and CD161 expression. Cell surface expression was analyzed on a FACS Calibur (BD Bioscience). Cell sorting was carried out using FACS ARIA II (BD Bioscience). Data were analyzed using FlowJo™ Software Version 10.5.3 (Becton, Dickinson and Company).

### RNA Sequencing (RNA-Seq)

For RNA sequencing, stimulated MAIT cells and as control unstimulated MAIT cells of five different donors were FACS-sorted based on their TCR Vα7.2 and CD161 expression, yielding a purity of >98%. Cells were lysed in 1 mL TRIzol (Thermo Fisher), RNA was isolated using miRNeasy Mini Kit (Qiagen) and Maxtract High Density tubes (Qiagen), residual DNA was removed using Ambion TURBO DNA-free Kit (Thermo Fisher) and RNA cleaned up by ethanol-precipitation. RNA concentration was determined by Qubit 2.0 instrument using the Quant-iT RNA kit (Thermo Fisher Scientific). The RNA integrity for each sample was controlled with the RNA 6000 Nano Assay and the Agilent 2100 Bioanalyzer (Agilent Technologies). All samples included in the experiment had RIN >8, and 100ng total RNA was used for rRNA depletion. Ribosomal RNAs were removed from total RNA using the Ribo-Zero Gold H/M/R Magnetic Kit (Illumina). A strand-specific library for transcriptome sequencing was prepared using the ScripSeqv2 Kit (Illumina), which was checked by Agilent 2100 Bioanalyzer system with a High Sensitivity DNA Kit (Agilent). Library concentration was determined by Qubit 2.0 instrument using the Quant-iT dsDNA High Sensitivity kit (Thermo Fisher Scientific). 10 ng from each library was pooled. Library pool was size-selected in a range of 150-700 bp using preparative agarose gel in combination with MinElute Gel Extraction Kit (Qiagen). Paired-end sequencing with 160 Mio read pairs per sample was performed at a length of 100 bases on HiSeq2000 (Illumina).

### Evaluation of Transcriptomic Data and Statistical Analysis

#### Generation of Read Counts

The workflow management system uap (20) was used to transform the paired-end sequencing reads into a quantitative presence/absence table. The RNA-Seq workflow, which is included in the software, was customized to the requirements of this analysis: Fastq files were merged followed by a quality control with FastQC (21) and a quality filter using the FASTQ Quality Filter of the FASTX-toolkit (22). Trim Galore (23) was employed for adapter trimming. The reads were mapped to the human genome (GRCh38) with HISAT2 (24). The samtools suite of programs (25) was used filter the alignments for proper pairing and for sorting them by name for subsequent processing with htseq_count (26) to generate counts of reads that overlap annotated genes. As a reference the GENCODE gene annotation (27) (Release 34) was used.

#### Differential Expression Analysis

To test for differential expression in the count data, the DESeq2 R package (28) (release 1.22.1) provided at Bioconductor, was used. The quality of the count data was assessed by exploring the sample-to-sample distances (data not shown) and a principal component analysis (PCA) of the samples. DESeq2 relies on Generalized Linear Models, Empirical Bayes Shrinkage of the log fold change and a Wald test on the model coefficients to assess differential expression. The variables used for modeling were defined in the design as treatment, and contrast fits were then computed for the two different treatment groups *vs* the unstimulated control group. To account for multiple testing, p-values where adjusted according to Benjamini-Hochberg (29). Genes were defined as differentially expressed if their adjusted p-value (p.adj) was ≤0.01.

#### Pathway Enrichment Analysis

Pathway alterations were investigated using differentially expressed genes (p.adj ≤0.01) in the Ingenuity Pathway Analysis (IPA) tool (Qiagen) (30, 31). Pathway enrichment was conducted using the immune cell database, and pathways with a Benjamini-Hochberg corrected p-value (p.adj) ≤0.05 were considered significantly enriched. Pathways with an IPA z-scores were evaluated.

#### Biological Network Inference

Weighted gene correlation networks were retrieved from the count data using the R package WGCNA (32, 33) (release 1.6.8). Lowly expressed genes, i.e., sum of reads per gene below 10 across all samples, were removed from the data set. Information regarding the treatment groups and time points was binarized. A soft-threshold β=10 was selected according to the soft-thresholding procedure in the WGCNA package. A signed network was constructed with minimal module size of 30 and a mergeCutHeight of 0.25. The correlation between resulting modules and the clinical traits was computed as the correlation between the module Eigengenes and the trait information and evaluated with a Student asymptotic p-value. Modules that jointly correlated to either a-CD3/a-CD28- or *E. coli*-treatment group *and* had correlation values above 0.4 (and resp. p-values below 0.04) were merged. Genes were annotated using Bionconductors’ biomaRt R package (34, 35) [with GENCODE gene annotation (Release 34)] and differential expression information were mapped. Gene significance (GS) and module membership (MM) were computed, and for the top 20 genes, according to those values, the adjacency information was extracted from the topological overlap matrix. Respectively annotated node and edge files were generated and visualized in Cytoscape 3.7.1 (36). RNA-Seq data are available at the Gene Expression Omnibus (GEO) accession number GSE158439. Data obtained for identified transcription factors and cytokines are summarized in **Supplementary Tables 1** and **2**, respectively.

### Cell Lysis, Protein Digestion, LC-MS/MS for Proteomics

For protein extraction, cells were washed 3 times in PBS and lysed in 8M Urea Buffer. Protein concentrations were determined using Pierce 660nm Protein Assay (Thermo Fischer Scientific, Germany). For global protein analysis, an untargeted proteomics approach was applied. Starting from 10 µg protein per sample, the volume was adjusted to the same volumes with 100mM Triethylammonium bicarbonate buffer (TEAB, Sigma Aldrich, Germany). Proteins were reduced with 0.1 µmol tris(2-carboxyethyl)phosphine (Sigma Aldrich, Germany) for 1 h at 55°C, followed by alkylation with 0.2 µmol iodoacetamide (Merck, Germany) for 30 min at room temperature in the dark. Afterwards, protein solutions were acidified, and acetonitrile (Merck, Germany) was added to reach more than 50% (v/v) organic content to facilitate protein binding to SpeedBeads™ magnetic carboxylate modified particles (SP3 beads, Sigma Aldrich, Germany), which allow for protein clean-up, digestion and peptide clean-up in one tube. The samples were processed on the beads as described before (37, 38) with minor adjustments. In brief, for each sample 2 µl of bead solution, containing 20 µg beads was used. Proteins were loaded on the beads, followed by washing with 70 % (v/v) ethanol (Merck, Germany) in water and then with 100 % (v/v) acetonitrile (ACN, Carl Roth, Germany). Next, proteins were digested with trypsin (enzyme:protein ratio 1:40, Promega, USA) in 100 mM TEAB in the same tube. Digestion was stopped by addition of 100 % (v/v) acetonitrile to reach ≥ 95 % (v/v) organic content, facilitating peptide binding to the beads. Peptide clean-up was performed using 100 % (v/v) acetonitrile. Finally, peptides were eluted in two steps, first with 87 % (v/v) ACN in ammonium formate (pH 10) (Agilent Technologies, USA), then with 2 % (v/v) dimethyl sulfoxide (Sigma Aldrich, Germany), thus resulting in two fractions per sample that were evaporated to dryness and reconstituted in 0.1% (v/v) formic acid (FA).

Both fractions were analyzed on a UPLC system (Ultimate 3000, Dionex, USA), coupled to a Q Exactive HF (Thermo Scientific, USA) as described previously (39). First, peptides were collected on a trapping column (Acclaim PepMap 100 C18, 3 µm, nanoViper, 75 µm × 5 cm, Thermo Fisher, Germany) at flow rate 5 µl/min using a solution of 2 % (v/v) ACN and 0.05 % (v/v) trifluoroacetic acid (TFA) in water. Subsequently, peptide separation on a reversed-phase column (Acclaim PepMap 100 C18, 3 µm, nanoViper, 75 µm × 25 cm, Thermo Fisher, Germany) was applied using a non-linear gradient of 150 min starting at inorganic conditions (0.1 % FA in water) and increasing proportion of organic solution (80 % ACN, 0.1 % FA in water). A chip-based ESI source (Nanomate, Advion, USA) was used for ionization at 1.7 kV and was coupled to the Q Exactive HF. The MS1 scans were acquired at a resolution of 120K in a range of 350–1550 *m/z*. AGC target was set to 3×10^6^ with a maximal injection time (IT) of 100 ms. The top 10 most abundant peptides were isolated for MS2 acquisition with an isolation window of 1.4 *m/z*. Peptides were fragmented at a normalized collision energy (NCE) of 28 and the fragment ion spectra were acquired at a resolution of 15K using AGC target of 2×10^5^ and maximal IT of 100 ms. All spectra were acquired using XCalibur (Version 3.0).

### Evaluation of Proteomic Data and Statistical Analysis

MS raw data were processed with MaxQuant Version 1.6.2.10 with the integrated search engine Andromeda using the MAXLFQ algorithm (40). If not stated otherwise, default parameters were used. Peptides were identified using a database search against the *Homo sapiens* UniprotKB reference proteome (2018-11, reviewed and unreviewed entries). Carbamidomethylation of cysteine was set as fixed modification, whereas oxidation of methionine and acetylation of protein N-termini were set as variable modifications. Peptide and fragment mass tolerance were set to 10 ppm (MS1), and 20 ppm (MS2). Minimum peptide length was set to 7. Peptide spectrum matches were evaluated using the target-decoy method with false discovery rate (FDR) ≤0.01. A minimum of two peptides with at least one unique peptide were required for protein inference again applying FDR ≤0.01. Match between runs was activated. Proteins were quantified based on unique and razor peptides, resulting in protein abundances for 51711 peptides and hence 4653 protein groups. MaxQuant quality control was carried out by PTXQC package^37^ in the R environment (data not shown). Protein contaminants and reverse hits were excluded using Perseus 1.6.2.3.

Protein intensities were processed, and results were visualized in R-3.5.0 using following packages: limma (41), plyr (42), reshape2 (43), xlsx (44), DEP (45), ggsci (46), circliz (47), calibrate (48), ggplot2 (49), ComplexHeatmap (50), dendsort (51), dendextend (52), pheatmap (53), readxl (54), qpcR (55), splitstackshape (56), tidyr (57), and Tmisc (58). Thus, the data were log2-transformed, filtered for proteins that were quantified in three replicates under at least one condition, followed by variance-stabilization. Imputation was performed using the DEP package (45) (fun = “MinProb”, q = 0.01) in the case of proteins that were not quantified in any of the replicates under the particular condition. Subsequently, fold changes (FCs) and p-values using Student’s t-test were calculated relative to the unstimulated control. Proteins with a p-value ≤0.05 were considered significantly altered.

Ingenuity Pathway Analysis, using significantly altered proteins (p-value ≤0.05), was conducted analogous to RNA-Seq data analysis.

For WGCNA, the log2-transformed, filtered, variance-stabilized and imputed data were used as described before (59) with minor differences. The networks were constructed across all the measured samples with the R package WGCNA (32), creating a signed network. The soft-threshold for WGCNA was set to β=9, and the Topology Overlap Matrix (TOM) was created using a mergeCutHeight of 0.25 and a minimum module size of 50. The analysis identified 9 modules of co-abundant proteins, assigned to different colors, and correlations of module Eigengenes with traits were examined using Pearson correlation and Student asymptotic p-values. Also the identification of trait-specific key drivers was performed based on the WGCNA results as described before (59). For this purpose, module- and trait-specific protein significances (PS) and module memberships (MM) were calculated for each analyte, and key drivers were assumed to be proteins with absolute PS ≥ 0.5 and absolute MM ≥ 0.5. Proteomics data are available in **Supplementary Table 3**.

### Cell Lysis, Extraction of Metabolites, LC-MS/MS for Metabolomics

For the extraction of intracellular metabolites, the medium was completely removed, and cells were quenched 3 times with 1 ml ice-cold 0.9 % sodium chloride. After removing the quenching solution, cells were resuspended in 100 µl ice-cold ACN followed by 100 µl ice-cold Milli-Q water. After vortexing for 1 min, cells were centrifuged (14000 rpm, 4°C, 10 min). Supernatants were transferred to new tubes. Intracellular metabolites were extracted again with 500 µl Methanol : ACN:Milli-Q water (2:3:1). After centrifugation (14000 rpm, 4°C, 10 min), both supernatants were combined, evaporated to dryness and stored at -80°C until measurement. Prior to analysis, all samples were resuspended in 25 µl 0.1% FA and 5% ACN in water.

For LC-MS/MS measurement, 10 µl of each resuspended extract was injected onto a HPLC system coupled online with a 6540 UHD Accurate-Mass Q-TOF (Agilent Technologies). Metabolites were separated on a Waters Acquity UPLC^®^ HSS T3 column (2.1 x 100 mm, 1.8 µm) equipped with a Waters Acquity UPLC^®^BEH C18 pre-column (2.1 x 50 mm, 1.7 µm). The autosampler and column oven were kept at 5°C and 45°C respectively. Separation was achieved with a binary solvent system (A: 0.1% FA in water and B: 0.1% FA in 50:50% ACN : MeOH), which was run with the following gradient: 0-5 min: 5% B; 5-19 min: 5-95% B; 19-21 min: 100% B; 21-21.5 min 100-5% B; 21.5-24 min: 5% B. Metabolites were eluted at a constant flow rate of 0.3 ml/min. Eluted metabolites were measured with the QTOF operated in centroid mode. Full scan data was generated with a scan range of 60-1600 *m/z* in positive ionization mode. Out of the survey scan, the 5 most abundant precursor ions with charge state = 1 were subjected to fragmentation. The dynamic exclusion time was set to 30 s.

### Evaluation of Metabolomic Data and Statistical Analysis

MS raw files (.d files) of all 20 samples, 4 pooled samples and 2 blank runs were imported into Progenesis QI (Non-Linear Dynamics) software. Data were analyzed in a generic workflow. The adduct ions involved [M+H], [M+H-H_2_O], [M+H-2 H_2_O] and [M+H+ACN]. First, chromatogram alignment was performed. The most suitable reference chromatogram was chosen automatically. The following software-guided peak picking tool resulted in a data matrix including the retention time, mass-to-charge ratio and corresponding normalized peak area. The matrix, referred to as feature list, was the basis for the subsequent automated database search. The ChemSpider plug-in was used as identification method with HMDB and KEGG as database resources.

After exporting the final results, the feature list was validated based on the 4 pooled samples, which were included in the measurement. Solely features with less than 30% variation in these samples remained for further analysis. Blank subtraction was done afterwards. Additionally, only features with more than 40% valid values within the 20 samples were considered for quantification resulting in 389 features. Metabolomics data are available in the **Supplementary Table 4**.

Metabolite intensities were processed, and results were visualized in R-3.5.0 using following packages: limma (41), plyr (42), reshape2 (43), xlsx (44), DEP (45), ggsci (46), circliz (47), calibrate (48), ggplot2 (49), ComplexHeatmap (50), dendsort (51), dendextend (52), pheatmap (53), readxl (54), qpcR (55), splitstackshape (56), tidyr (57), and Tmisc (58). According to the proteomic data, the metabolomics data were log2-transformed, filtered for metabolites that were quantified in three replicates under at least one condition, followed by variance-stabilization. Imputation was also performed using the DEP package (45) (fun = “MinProb”, q = 0.01) in the case of metabolites that were not quantified in any of the replicates under the particular condition. Subsequently, fold changes (FCs) and p-values using Student’s t-test were calculated relative to the unstimulated control. Metabolites with a p-value ≤0.05 were considered as significantly altered. The metabolome data are available at the Metabolomics Workbench repository (DOI:10.21228/M8T69T).

### Quantitative Real-Time PCR

For quantitative real-time PCR (qPCR), RNA isolation and reverse transcription was done as described previously (60). Briefly, RNA was isolated using the RNeasy Mini kit with DNase I digestion (both Qiagen, Germany). 500ng RNA was used to synthesize cDNA using the High-Capacity cDNA Reverse Transcriptase Kit (Applied Biosystems, USA). The qPCR was performed using TaqMan™ Fast Advanced Master Mix (Applied Biosystems, USA) on a qPCR ABI 7500 FAST Real-Time PCR System (Applied Biosystems, USA) using TaqMan™ primer sets for human TWIST1, MTHFD2 and SDHA (Applied Biosystems, USA). Gene expression was measured by the ΔΔ_ct_-Method relative to the unstimulated control and with SDHA as references. Levels of significance were determined by Student’s t-test in GraphPad Prism 9.

### Measurement of Cytokine Production

Cytokine release was determined using the LEGENDplex™ human CD8/NK Panel from cell culture supernatants of isolated MAIT cells following the manufacturer’s instructions. Cytokine release was analyzed on a FACS Calibur (BD Bioscience). Median fluorescence intensities (MFI) were interpolated using an asymmetric sigmoid 5PL standard curve in GraphPad Prism resulting in absolute concentrations in pg/ml. Levels of significance were determined by Student’s t-test in GraphPad Prism 9.

## Results

### Transcriptome Profiling of Activated Human MAIT Cells

**Figure 1:**
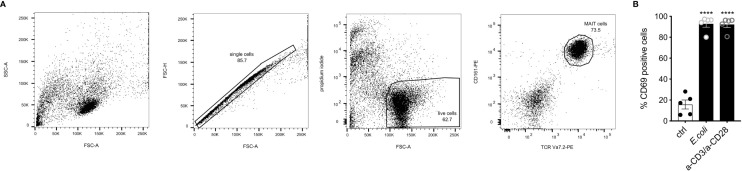
Gating strategy for fluorescence-activated cell sorting prior RNA-sequencing. **(A)** Gating strategy for fluorescence-activated cell sorting to identify human MAIT cells, after co-culture with monocytes followed by RNA-Seq. First, cells were gated on single cells (FSC-A *vs*. FSC-H), followed by gates on live cells (FSC-A *vs*. propidium iodide). MAIT cells were defined as TCR Va7.2+ CD161+ double-positive cells. Representative dot plots from one of five donors are displayed. **(B)** Activation of MAIT cells by *E.coli*-loaded monocytes and a-CD3/a-CD28 antibodies determined by CD69 cell surface expression (n=5 ± SEM).

Robust activation of MAIT cells during infection has been shown to depend on both antigen-dependent and -independent signals (10, 61). The induced molecular profile highly depends on the mode of activation (19). *E. coli*-induced activation was shown to induce such a robust activation in a TCR-dependent and -independent manner (14, 19). To characterize the transcriptome of a robust human MAIT cell activation, we activated the MAIT cells for 16 h using *E. coli*-pulsed monocytes. For comparison to a pure TCR-induced activation, MAIT cells were activated using anti-CD3/anti-CD28 antibodies. MAIT cells were sorted based on their TCR Vα7.2 and CD161 expression after 16h of stimulation (**Figure 1A**) and processed for paired-end RNA-Seq. The surface expression of the early activation marker CD69 was determined by flow cytometry. As described previously (14), stimulation of MAIT cells by fixed *E. coli* led to a consistent and strong activation as revealed by CD69 expression comparable to stimulation by anti-CD3/anti-CD28 antibodies (**Figure 1B**).

**Figure 2:**
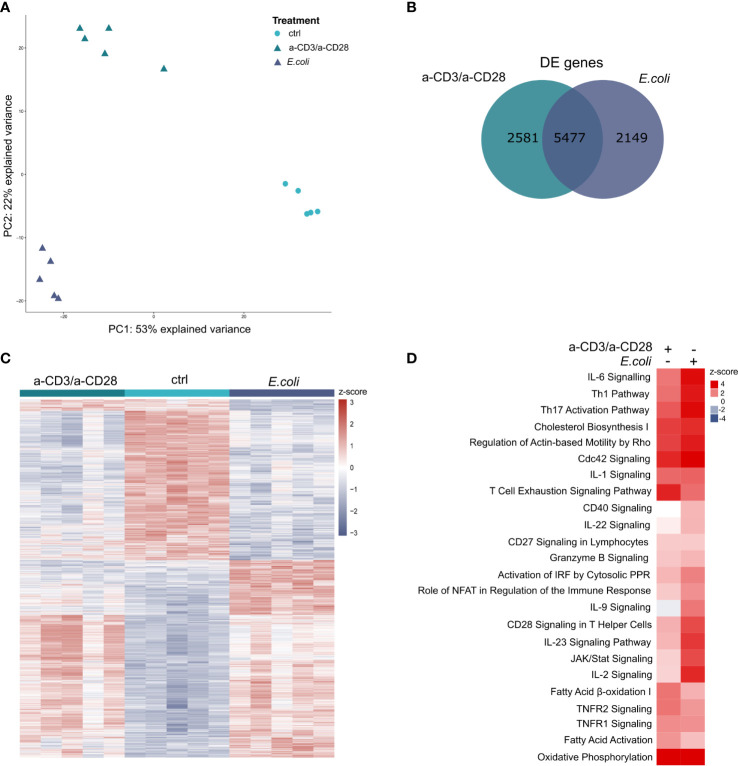
Transcriptomic profiling of activated human MAIT cells **(A)** Principal component analysis of activated MAIT transcriptomes. Each symbol represents one donor (n=5). **(B)** Overlap of totally expressed and differentially expressed genes (p.adj ≤ 0.01) in MAIT cells stimulated either with *E. coli*-pulsed monocytes or anti-CD3/a-CD28 antibodies or left untreated (ctrl). **(C)** Hierarchical clustering and heatmap of differentially expressed genes. **(D)** Enriched core pathways of DE genes (p.adj ≤ 0.01) during MAIT cell activation determined by IPA (p.adj ≤ 0.05, z-scores).

In total, we found 36,484 annotated genes expressed. The variance in the expression data can be mainly explained by the treatment (1^st^ principal component (PC) with 53%) and the mode of activation (2^nd^ PC with 22%), indicating a clear separation of the differently treated samples (**Figure 2A**). The analysis of differentially expressed (DE) genes between stimulated cells and unstimulated controls revealed 8,058 DE genes between a-CD3/a-CD28 activated cells and unstimulated controls, and 7,626 between *E. coli*-stimulated cells and unstimulated controls, respectively. A total of 5,477 genes was DE in both contrasts, with 99% (5,397) regulated in the same direction (**Figure 2B**). The 2,581 selectively regulated DE genes for a-CD3/a-CD28-activated cells include 1,948 protein-coding genes and 127 lincRNAs. Among the 2,149 selectively regulated DE genes for *E. coli*-stimulated cells were 1,660 protein-coding and 104 lincRNAs. Hierarchical clustering of differentially expressed genes revealed a specific pattern for each type of activation (**Figure 2C**).

Next, we analyzed the subset of DE genes that code for proteins (*E. coli*-stimulated: 6,056; a-CD3/a-CD28 activated: 6,344) for significantly enriched pathways during activation using Ingenuity Pathway Analysis (IPA). The analysis yielded in total 184 enriched pathways with an IPA z-score, reflecting an up- or down-regulation of the corresponding pathway (**Supplementary Figure 1**). Upon activation with fixed *E. coli*, or a-CD3/a-CD28 antibodies, MAIT cells significantly upregulated pathways related to immune functions like various interleukin (IL) signalling pathways, JAK/STAT Signalling, Role of NFAT, Granzyme B Signalling, and metabolomic pathways, i.e., Cholesterol Bioysynthesis I and Fatty Acid Activation (**Figure 2D**). As expected, the Th1 Pathway, Th17 Activation Pathways and Granzyme B Signalling were upregulated by both activations, with a stronger response in MAIT cells activated by *E. coli*, than by the TCR-stimulus alone (**Figure 2D**).

### Transcriptional Regulation of MAIT Cell Activation

**Figure 3:**
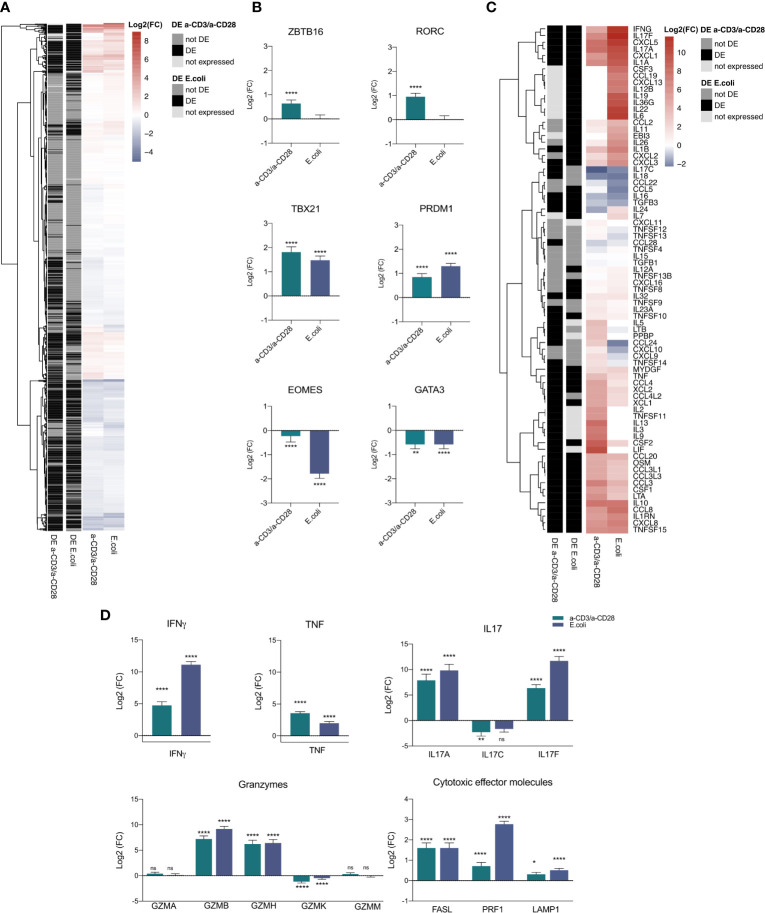
Transcription factor and cytokine mRNA profile of activated MAIT cells. **(A)** Heatmap of 1115 expressed transcription factors in the transcriptome of a-CD3/a-CD28 or *E.coli*-activated MAIT cells. **(B)** Log2(FC) mRNA expression of selected transcription factors (mean +SD, n = 5). Levels of significance are given as FDR-adjusted p-values with ****≤0.0001,***≤0.001, **≤0.01 and *≤0.05. **(C)** Heatmap of 75 expressed cytokines and chemokines in the transcriptome of a-CD3/a-CD28 or *E.coli*-activated MAIT cells. **(D)** Log2(FC) mRNA expression of selected cytokines and cytotoxic molecules in activated MAIT cells (mean +SD, n = 5). Levels of significance are given as p.adj with ****≤0.0001, ***≤0.001, **≤0.01 and *≤0.05; n.s., not significant.

After activation, the fate of T cells is mainly determined by transcription factors (TFs). Hence, to illuminate the underlying transcriptional mechanisms of MAIT cell activation, we screened the expression profiles of activated MAIT cells for TFs based on the 1,639 known and likely TFs published by Lambert et al. (62). We found 1113 TFs within the dataset (**Figure 3A**, **Supplementary Table 1**), with a total of 369 TFs being DE in both contrasts and 99% (364) regulated in the same direction. While 205 TFs were DE in a-CD3/a-CD28-activated MAIT cells, 117 were found DE only in *E. coli*-activated MAIT cells (**Figure 3A**). The TFs that were previously described for MAIT cells, i.e., *RORC, ZBTB16/*PLZF*, TBX21/*T-bet (63), *EOMES*, *PRDM1*/BLIMP-1 (19) and *GATA3* (64) were expressed in our data as well. However, depending on the mode of activation, expression of those TFs varied (**Figure 3B** **)**. While EOMES and GATA3 were significantly down-regulated in *E. coli*-activated MAIT cells, only GATA3 was down-regulated in TCR-stimulated MAIT cells (**Figure 3B**). *TBX21/*T-bet, *ZBTB16/*PLZF, *PRDM1*/BLIMP-1 and *RORC* were upregulated in TCR-stimulated MAIT cells, while in *E. coli*-activated MAIT cells only *TBX21/*T-bet and *PRDM1*/BLIMP-1 were significantly upregulated. Taken together, our results confirm that changes in the transcription factor profile after 16 h depend on the way of activation in human MAIT cells.

### Effector Molecule Profile During MAIT Cell Activation

In bacterial and viral infections, MAIT cells have been shown to produce cytotoxic effector molecules and pro-inflammatory cytokines as well as chemokines after activation, depending on their mode of activation (19). Since these effector molecules are highly regulated on the transcriptional level, we investigated their transcription pattern within the transcriptome of activated MAIT cells. Based on 133 cytokine and chemokine genes published by Pro et al. (65), we found 76 genes expressed in our data set (**Figure 3C**). We ranked these cytokines and chemokines based on high, medium and low expression (**Supplementary Figure 2**), indicating relevant expression levels of all differentially expressed genes. Activation by *E. coli* led to a different cytokine pattern than a-CD3/a-CD28 stimulation (**Figure 3C** and **Supplementary Table 2**).

The classical MAIT cell cytokines IFNγ, IL-17A and IL-17F were highly upregulated in both treatments with a stronger response in MAIT cells activated by *E. coli* compared to stimulation with anti-CD3/anti-CD28 antibodies **(** **Figure 3D**), suggesting that activation by *E. coli* triggers additional pathways than TCR activation alone. TNF, in contrast, was stronger upregulated by TCR stimulation alone **(** **Figure 3D**).

To define the cytotoxic effector molecule repertoire of MAIT cells, we screened the RNA-Seq dataset for granzymes and found all 5 human granzymes *GZMA, GZMB, GZMK, GZMM* and *GZMH* expressed in both a-CD3/a-CD28- and *E. coli*-activated MAIT cells. Again, the two ways of activation induced a specific pattern. *GZMA* and *GZMM* were both expressed in a-CD3/a-CD28- and *E. coli*-activated MAIT cells, without significant differences. *GZMB* and *GZMH* were significantly up-regulated in both treatments, while *GZMK* was down-regulated in both treatments, but only significantly during a-CD3/a-CD28 activation (**Figure 3D**). FASL, Perforin and *LAMP1/*CD107a were significantly up-regulated in both treatments (**Figure 3D**), with gene expression of *LAMP1/*CD107a stronger induced in *E. coli*-activated MAIT cells.

In conclusion, our data suggest that MAIT cells respond to *E. coli*-induced activation with a specific RNA expression profile of effector molecules that differs from the profile induced by TCR-dependent activation, thus again confirming the dependency of the MAIT effector function on the mode of activation.

### Identification of Key Genes Involved in the Distinct Modes of Activation

**Figure 4:**
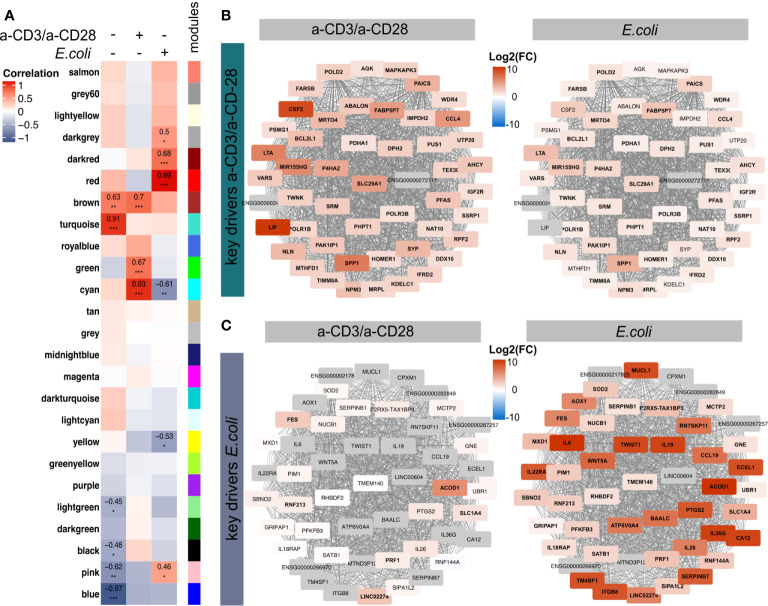
Weighted Gene Correlation Network Analysis of the transcriptome of activated MAIT cells. **(A)** Module trait correlation plot showing 25 by color-labeled modules, containing co-expressed genes, that were correlated to the three treatments. Correlation coefficients were added as numbers and corresponding p-values as asterisks with ***≤0.001, **≤0.01 and *≤0.05. The top 50 genes with highest intramodular connectivity and gene significance, reflecting putative key drivers (hub genes) for **(B)** a-CD2/a-CD28 and **(C)** *E. coli*-activated MAIT cells, were determined. Log2(FC) of a-CD3/a-CD28 or *E.coli*-activated MAIT cells compared to unstimulated controls are displayed with significantly regulated genes (p.adj ≤0.01) in bold. Genes in grey were only quantified in activated samples, thus Log2(FC) is not available.

Next, we aimed to identify relevant molecules defining the distinct expression signatures in the two ways of activation. Hence, we performed a Weighted Gene Correlation Network Analysis (WGCNA) (38), offering a differential-expression-independent analysis suitable to identify novel key drivers of biological treatments (32, 38, 59, 66, 67). Considering all the 36,484 quantified transcripts from the two treatment groups and the control group, we obtained 25 modules, containing genes with similar expression pattern, named by colors (**Figure 4A**, **Supplementary Figure 3A**). By correlating these modules to the two treatments and the unstimulated control, we identified 15 modules with significant correlations (**Figure 4A**). Since we were particularly interested in the differences between both modes of activation, *E. coli* and a-CD3/a-CD28, we merged all significant modules with a correlation > 0.4 for the respective treatment (**Supplementary Figure 3B**). As a result, we obtained two modules with 5,857 and 1,263 genes, respectively, named by the corresponding treatment, *a-CD3/a-CD28* and *E. coli*, respectively (**Supplementary Figure 3C**). Pathway enrichment analysis of all genes within these modules showed a clear enrichment of Th1- and Th17-related pathways and cytokine-signalling-induced pathways for the *E. coli*-specific module (**Supplemetary Figure 3D**). In contrast, pathways related to Sirtuin Signalling and Mitochondrial Dysfunction were enriched in the a-CD3/a-CD28 specific module (**Supplementary Figure 3D**). We further identified the 50 key genes showing the highest intramodular connectivity (module membership, MM) and gene treatment significance (GS) (**Figures 4B, C**, **Supplementary Figure 3E**). Among the key drivers of a-CD3/a-CD28 activation cytokines were substantially increased, including *LIF* and *CSF2* among others (**Figure 4B**). Expression of these key genes showed weaker expression levels in *E. coli-*activated MAIT cells. However, most of them were also significantly induced in *E. coli*-activated MAIT cells, reflecting the TCR-dependent regulation present in both ways of activation.

In contrast, *E. coli* activation led to a different pattern of key genes with a sharp upregulation of, e.g*., CD36G, IL26, IL6, CCL19*, or *PRF1/*Perforin, which were not induced by a-CD3/a-CD28 activation. This demonstrates additionally TCR-independent mechanisms induced by *E. coli*-mediated activation. Interestingly, most of the key genes, including cytokines, showed high connectivity to the transcriptional regulator TWIST1 (**Figure 4C**). TWIST1 was not induced in a-CD3/a-CD28 activated cells, suggesting an important role in *E. coli*-mediated activation of MAIT cells.

### The MAIT Proteome During TCR-Dependent and -Independent Activation

The transcriptomic data presented herein suggest a distinct molecular signature depending on the mode of activation, which was also described by Lamichhane et al. (19). However, it is still unclear if these differences on the transcriptomic level are also relevant for the phenotype that can be analyzed by investigating the proteome. Thus, and to further discriminate between TCR-dependent and -independent activation, we used a global proteomics approach and stimulated MAIT cells *in vitro* with anti-CD3/CD28 antibodies and IL-12/IL-18, respectively or a combination of both. Using high-resolution liquid chromatography-tandem mass spectrometry (LC-MS/MS), we identified 4,203 protein groups within samples from 4 donors. Overall, we quantified 3,002 proteins in 3 of 4 replicates.

**Figure 5:**
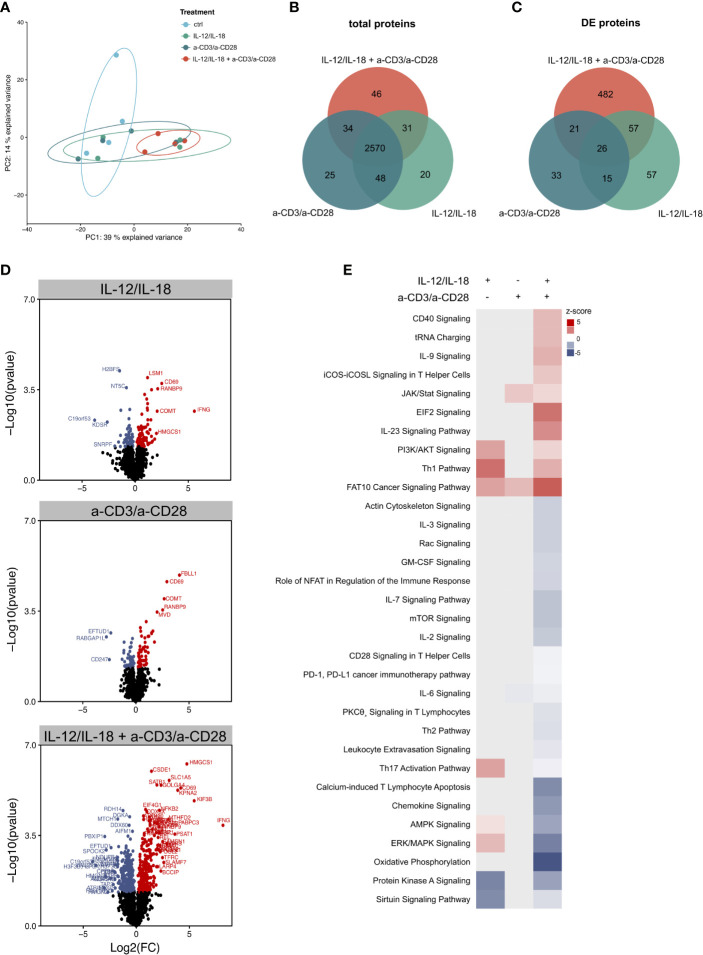
Proteomic profiling of MAIT cell activation. **(A)** Principle component analysis of the proteome of MAIT cells stimulated with IL-12/IL-18, a-CD3/a-CD28 or a combination of both. Each point represents a single donor (n=4). Overlap of **(B)** all detected proteins and **(C)** differentially abundant proteins in stimulated MAIT cells. **(D)** Volcano plots highlighting differentially abundant proteins in stimulated MAIT cells (p-value ≤ 0.05). **(E)** Heatmap of selected, significantly enriched pathways with a z-score reflecting up- or downregulation, determined by IPA (padj ≤ 0.5).

Interestingly, the principal component analysis revealed only an insufficient separation of IL-12/IL-18 or a-CD3/a-CD28-stimulated MAIT cells, but a strong separation of the combined treatment, compared to unstimulated cells (**Figure 5A**). 2,570 proteins were jointly expressed in all 3 ways of activation, while a minor number of proteins (20–46) were selectively expressed after the different treatments **(** **Figure 5B** **)**. Analysis of significantly altered proteins revealed that more proteins were regulated in a-CD3/a-CD28 (TCR-dependently)- than IL-12/IL-18- (TCR-independently) activated cells (**Figure 5C**). Most changes in protein abundance occurred when cells were treated with a combination of TCR stimulus and cytokines (**Figure 5D**). Analysis of enriched pathways by IPA again showed the strongest changes in MAIT cells, stimulated with a combination of cytokines and TCR-stimulus compared to single treatments (**Figure 5E**). Interestingly, the Th1 pathway was upregulated, while the Th17 pathway was downregulated in the combined treatment. Notably, cytokine-activation alone induced an upregulation of both Th1 and Th17 pathways. Overall, these results show that TCR-dependent and -independent activation led to distinct molecular effects on the pathway level after 16h of stimulation in human MAIT cells on the proteome layer.

**Figure 6:**
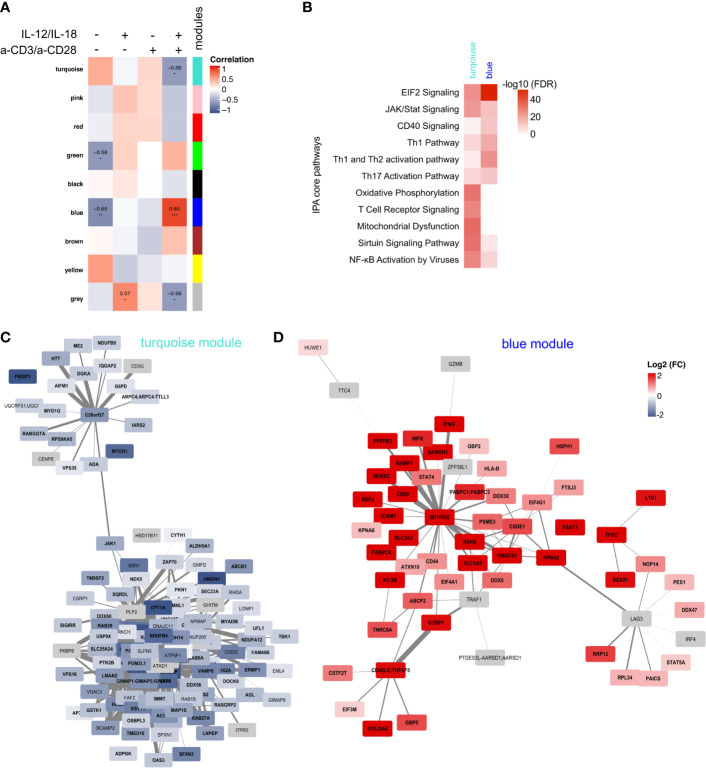
Weighted Gene Correlation Network Analysis of the proteome of activated MAIT cells. **(A)** Module trait correlation plot showing 25 by color-labeled modules, containing co-abundant proteins, that were correlated to the four treatments. Correlation coefficients were added as numbers and corresponding p-values as asterisks with ***≤0.001 **≤0.01 and *≤0.05. **(B)** IPA enrichment of proteins from the blue and the turquoise module. Key driver proteins of activated MAIT cells from the modules turquoise **(C)** and blue **(D)** with absolute MM and PS ≥0.5 and connectivity threshold of ≥0.25. Log2(FC) of IL-12/IL-18 + a-CD3/a-CD28-activated MAIT cells compared to unstimulated controls are displayed with significantly regulated proteins (p-value ≤0.05) in bold. Proteins in grey were only quantified in activated samples, thus Log2(FC) is not available.

To identify key proteins involved in TCR-dependent and TCR-independent activation of MAIT cells also on the proteome level, we again applied WGCNA. We obtained nine modules that were correlated to the three treatments and the unstimulated control (**Supplementary Figure 4A**), resulting in three modules with a significant correlation with at least one treatment and one module with a significant correlation for two treatments (**Figure 6A**). Due to significant correlations with MAIT cells stimulated with a combination of cytokines and anti-TCR antibodies, we selected the blue and the turquoise module for further analysis, containing 539 and 941 proteins, respectively (**Supplementary Figure 4B**). IPA showed enrichment of core pathways involved in Th cell activation, JAK/STAT, CD40 and EIF2 Signalling for the blue module (**Figure 6B**), that were positively associated to activated cells and negatively to untreated controls (**Figure 6A**). In contrast, the turquoise module that negatively correlates with MAIT cell activation showed enrichment of pathways related to, e.g., TCR signalling, Oxidative Phosphorylation, or Mitochondrial Dysfunction (**Figure 6B**). Further, key proteins were identified based on their intramodular connectivity (module membership, MM) and protein trait significance (protein significance, PS). We considered proteins with an absolute MM and PS correlation ≥0.5 as key proteins with minimal connectivity of 0.25 (**Supplementary Figure 4C**). For the turquoise module, we obtained 121 putative key proteins, including CD3G, ZAP70 and JAK1, that were downregulated after activation (**Figure 6C**). For the blue module, key proteins involved in activation were strongly upregulated, e.g., CD69, CD71 (TFRC), REL, IFNγ, Granzyme B or CD40LG (**Figure 6D**). Interestingly, we could identify three sub-clusters showing central proteins with a high connectivity to other key drivers, i.e., MTHFD2, CD40L or LAG3. MTHFD2 was also identified as a key driver in the transcriptome of a-CD3/a-CD28-stimulated MAIT cells. Depending on the mode of activation, abundance of these three key regulators differed (**Supplementary Figure 4D**).

### Cross-Validation of MAIT Cell Activation by Transcriptome and Proteome

**Figure 7:**
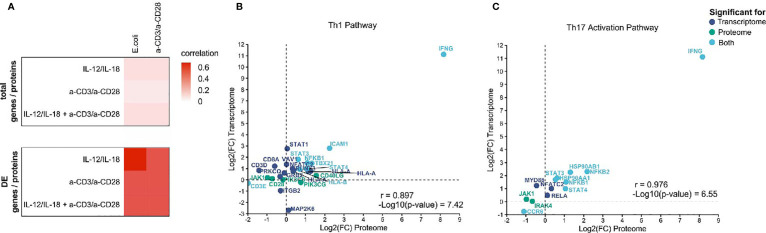
Integration of global mRNA and protein signatures. **(A)** Heatmap of correlation coefficients for total genes/proteins and differentially expressed genes/proteins in activated MAIT cells. Correlation of transcripts and proteins assigned to **(B)** the Th1 pathway and **(C)** the Th17 activation pathway from IPA. Displayed are only genes or proteins that were significantly altered in transcriptome (p.adj ≤ 0.01, dark blue), proteome (p-value ≤ 0.05, green) or both omics layers (light blue).

A multi-omics approach allows the cross-validation of investigated omics layers. Thus, and to analyze whether the integration of transcriptome and proteome adds additional insights regarding the mechanism of activation of MAIT cells, we correlated transcriptomic and proteomics data generated within this study. The correlation of proteome and transcriptome on the global level was moderate with correlation coefficients (r) between 0.2 and 0.3 (**Figure 7A**). However, considering only differentially expressed transcripts (p.adj ≤0.01) and proteins (p-value ≤0.05) revealed high correlation coefficients with the highest correlation for IL-12/IL-18 proteome/*E. coli* transcriptome (**Figure 7A**), indicating a substantial role of cytokine-mediated MAIT cell activation during bacterial infection.

Further, we correlated mRNA and protein profiles for those assigned to particular pathways based on IPA results, focusing on the Th1 and Th17 pathways. We found highly diverse correlation patterns between transcriptome and proteome for both pathways in the various treatments with r = 0 - 0.98 (**Supplementary Figure 5**). The most significant correlation was observed for both the Th1 and Th17 pathways comparing the transcriptome of *E. coli*-activated MAIT cells and the proteome of cytokine-activated MAIT cells (**Figures 7B, C**).

Overall, the results indicate that protein and mRNA signature differ at the single-molecule level but showed a similar direction for significantly regulated mRNAs or proteins and thus on the pathway level.

### The MAIT Metabolome During TCR-Dependent and -Independent Activation

Cellular metabolism has been shown to be a key regulator of immune cell function and consequently, regulating homeostasis and inflammation (68). Thus, we next analyzed the metabolome of TCR-dependent and -independent activated MAIT cells using an untargeted approach. Cells from 4 different donors were stimulated in accordance with the proteomics data and analyzed by high-resolution MS, resulting in 986 features and 389 identified metabolites (**Supplementary Table 4**). Principal component analysis showed only a minor separation of treatments and controls (**Supplemetary Figure 6A**). Interestingly, we observed a high overlap of all metabolites but only a minimal overlap of differentially abundant metabolites between the distinct treatments (**Supplementary Figures 6B, C**). Again, differentially abundance analyses revealed the most changes in MAIT cells stimulated with a combination of anti-CD3/CD28 antibodies and IL-12/IL-18 (**Supplementary Figure 6D** and **Supplementary Table 4**), which is in agreement with the proteomics results.

### The Role of Selected Key Drivers in MAIT Cell Activation

**Figure 8:**
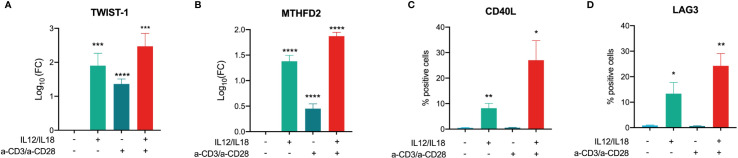
Validation of key driver expression in activated MAIT cells. Log10(FC) of **(A)** TWIST1 and **(B)** MTHFD2 mRNA in MAIT cells stimulated with IL-12/IL-18, a-CD3/a-CD28, or a combination of both (mean +SEM, n = 5). Cell surface expression of **(C)** CD40L and **(D)** LAG3 in MAIT cells stimulated with IL-12/IL-18 or a-CD3/a-CD28, or a combination of both were determined by flow cytometry using specific antibodies (mean +SEM, n = 4). Levels of significances are given as p-values with ****≤0.0001, ***≤0.001, **≤0.01, and *≤0.05.

Next, we validated selected key drivers in TCR-dependent and -independent activation. TWIST-1 and MTHFD2 mRNA expression were both increased in MAIT cell activation with stronger responses in TCR-independent activation (**Figures 8A, B**), The highest induction of gene expression was observed for MAIT cells treated with a combination of TCR-dependent and -independent activation for both investigated key drivers (**Figures 8A, B**). For CD40L and LAG3, we observed increased cell surface levels only when cells were stimulated with cytokines or cytokines combined with a-TCR antibodies (**Figures 8C, D**), thus confirming the omics results.

**Figure 9:**
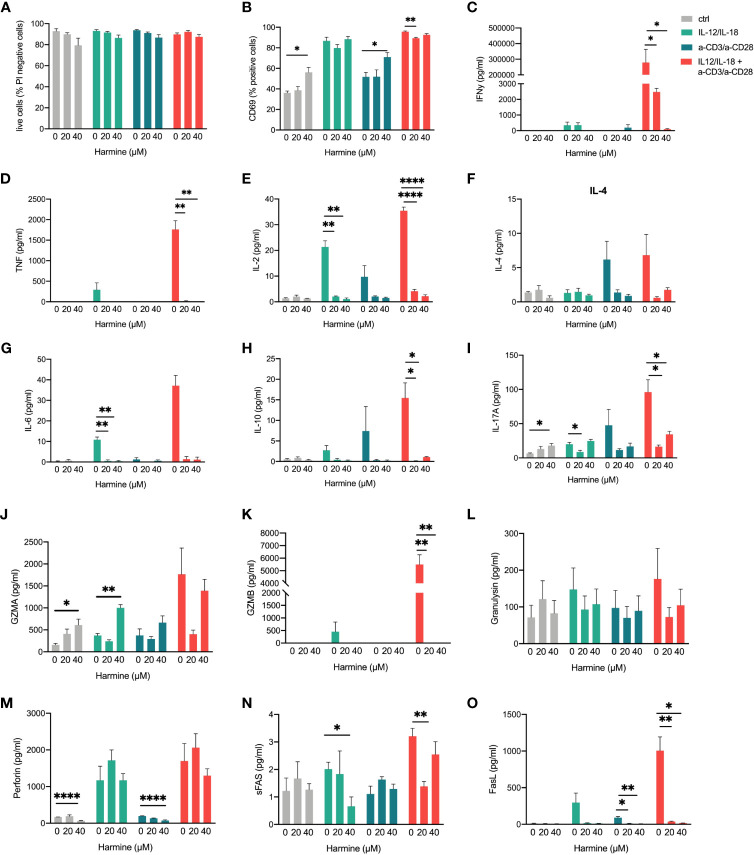
Effects of TWIST1 inhibition in activated MAIT cells. MAIT cells were stimulated with IL-12/IL-18, a-CD3/a-CD28, or a combination of both and **(A)** cell viability using propidium iodide staining, **(B)** CD69 cell surface expression using an anti-CD69 antibody as well as cytokine **(C–I)** or cytotoxic effector molecule release**(J–O)** using the LEGENDplex™ Human CD8/NK Panel bead assay were determined by flow cytometry (mean +SEM, n = 3). Levels of significances are given as p-values with ****≤0.0001, ***≤0.001, **≤0.01 and *≤0.05.

Finally, we evaluated the effects of TWIST1 inhibition on TCR-dependent and -independent activation in MAIT cells using the small molecule inhibitor harmine (69). Treatment with harmine did not significantly affect cell viability (**Figure 9A**), but we observed an increase in cell surface expression of the activation marker CD69 (**Figure 9B**). At the same time, cytokine release of almost all investigated 13 cytokines was affected in at least one of the treatments (**Figures 9C** **–I**). TWIST1 inhibition increased IL-17 and GZMA release of non-stimulated cells, while all other effects were associated to downregulation of effector-molecule release (**Figures 9J**–**O**), suggesting a supporting role in the activation of gene expression in MAIT cells.

## Discussion

MAIT cells are essential players in the early immune response, providing quick and efficient immune protection against bacterial and viral infections. In chronic inflammatory disorders, e.g., autoimmune and metabolic diseases, MAIT cells can contribute to maintaining the pathology, rendering them attractive targets for therapeutic approaches. Hence, mechanistic understanding of MAIT cell activation is of high relevance. Using a multi-omics approach with untargeted transcriptomics, proteomics and metabolomics, we provide in-depth insights into the mechanisms underlying the activation of human MAIT cells.

Pathway analysis of differentially expressed transcripts revealed that *E.coli* induced more substantial changes than TCR-stimulation on the pathway level. These variations may be explained by the distinct transcription factor expression of the mode of activation, showing that MAIT cells undergo a differential transcriptional reprogramming. Besides the described typical MAIT cell transcription factors *RORC, ZBTB16/*PLZF*, TBX21/*T-bet (63), *EOMES*, *PRDM1*/BLIMP-1 and *GATA3* (19), we found many other TFs differentially expressed, whose expression profiles varied between the two modes of activation. As an important consequence, we also observed differences in the effector function. Activated MAIT cells release pro-inflammatory cytokines and cytotoxic effector molecules like granzymes or perforin (14). In our study, we present a deep cytokine, effector molecule and chemokine profile of activated, circulating MAIT cells. We found that besides the already known cytokines IFNγ, TNF and IL-17, MAIT cells induce expression of a variety of cytokines, chemokines and effector molecules, whose relevance needs to be addressed in further studies. Depending on the mode of activation, these expression profiles differ, which is in accordance with Lamichhanne et al. (19), although different time points of activation were investigated. Moreover, our RNA-Seq data provide even deeper coverage of the transcriptome with more than 13000 protein-coding genes and hundreds of non-coding transcripts, whose relevance needs to be investigated in further studies. Moreover, by using a network-based analysis approach, we identified novel key genes, that are either up- or downregulated in distinct modes of activation. In *E. coli*-activated MAIT cells, the transcription factor TWIST1 was identified as key driver, showing a high connectivity to cytokine and chemokine expression. TWIST1 expression has been shown to be induced by signalling pathways linked to NfKB (70), NFAT (71), TCR (71) or STAT3 (72) and STAT4 (71). Signalling pathways related to those transcription factors, e.g., Th17 or cytokine signalling were both upregulated in our transcriptome and proteome data sets. However, TWIST1 expression was only upregulated in *E. coli*-stimulated cells, while no expression was detected in a-CD3/a-CD28 (TCR)-activated cells. Quantification of mRNA level confirmed that cytokine-induced activation has a higher effect on TWIST1 mRNA level than TCR-induced activation at 16h of stimulation. This suggests a regulation of TWIST1 expression by TCR-independent mechanisms in MAIT cells, or a different dynamic of TCR-induced expression. Considering the function, TWIST1 can act as both activator and repressor of gene expression and was shown to negatively regulate the T helper cell compartment (71, 73), particularly the Th17 development (74). In our study, inhibition of TWIST1 by a small molecule modulated the release of cytokines and effector molecules and the expression of the activation marker CD69. Interestingly, our results suggest that TWIST acts as an activator of most investigated cytokines and effector molecules since inhibition of TWIST1 decreased their release. This contrasts to effects observed in other T cell subset before (71), but MAIT cells are a distinct subset with distinct effector function. Moreover, previous studies focused on TCR-dependent effects of TWIST-1 function in T helper cells, while observed upregulation of TWIST1 mRNA in MAIT cells mainly depended on TCR-independent mechanisms. Hence, TWIST1-induced cellular mechanisms may be different in MAIT cells. Nevertheless, the utilization of small molecule inhibitors in primary human cells may have undesired side-effects, requiring investigation of the underlying molecular mechanisms resulting in the here observed TWIST1-dependent activation of gene expression that should be assessed in further studies.

While the transcriptomic data generated in this study give mainly information on the regulation of cellular processes, the proteome and the metabolome provide the most information on the phenotype. We thus aimed to analyze the phenotypic response of TCR-dependent and -independent activation of MAIT cells. So far, only global proteomic data of *ex vivo* and unstimulated MAIT cells (16) and no global metabolomic data of MAIT cells were available. Our study provides an in-depth proteomic and metabolomic profile of different modes of activated MAIT cells. The analysis revealed that most proteome and metabolome changes occur when MAIT cells are activated in a combination of TCR-dependent and -independent manner. To date, metabolomic data analysis is unfortunately limited due to the lack of valid identification of detected features, thus hampering conclusions on regulated metabolites. However, the here presented metabolomic data confirm that metabolome changes also depend on the mode of activation.

Nevertheless, by using a network-based analysis approach of the proteomic data, we identified novel key proteins, that are either up- or downregulated during activation. Proteins that were upregulated could be separated into three major clusters, with MTHFD2, CD40L and LAG3 as central proteins of each of those clusters.

The mitochondrial bifunctional methylenetetrahydrofolate dehydrogenase/cyclohydrolase MTHFD2 is a folated-coupled enzyme that was shown to be highly expressed in proliferating CD4+ T cells, and overexpression led to an increased proliferation of cancer cells (75). Activation-induced proliferation of MAIT cells has been shown earlier (14), and thus, MTHFD2 may serve as an important regulator in this process. In our data, MTHFD2 was particularly highly expressed in MAIT cells stimulated by a combination of TCR-dependent and -independent activation and showed a high co-expression with proteins relevant in activation, e.g., NFkB2, CD69 and IFN-γ. Therefore, MTHFD2 may also play a role in establishing effector function during robust activation of MAIT cells. In addition, MTHFD2 is induced downstream of mTOR complex 1 (76), which is essential to integrate immune signals from antigen-presenting cells, environmental cues, and nutrients and controls T cell fate (77).

The costimulatory molecule CD40L (CD154) was determined as a key connecting protein of the second cluster and was highly upregulated only by combined treatment, but not by TCR- or cytokine-stimulation alone. The relevance of CD40L expression in MAIT cells has been shown earlier (19, 78). During activation, MAIT cells upregulate CD40L, enabling them to mediate DC maturation (78). Moreover, CD40L expression reflects the potential in providing B cells (79). We observed a strong co-expression of CD40L with CSTF2T, EIF3M, GOLGA4 and GBP5, as well as G3BP1.

Lymphocyte-activation gene (LAG)-3, the key protein of the third cluster, was significantly more abundant only after combined TCR-dependent and independent activation. LAG-3 is a transmembrane receptor and has been shown earlier to be activated in T cells following TCR and/or cytokine stimulation, promoting IFN-y, TNF or IL-12 production (80, 81). LAG-3 is a co-inhibitory receptor, which binds to MHC class II on antigen-presenting cells in order to suppress further T cell activation and cytokine secretion, preventing excessive inflammation and facilitating a state of immune homeostasis (82). LAG3 expression in conventional T cells renders them susceptible to regulatory T cell subsets and affects differentiation (83). In patients with inflammatory bowel disease, LAG-3 expressing T cells are primarily found at sites of mucosal inflammation and their numbers correlate with disease activity (80). LAG-3 expression was accompanied by high IFN-γ concentration in activated MAIT cells, as previously shown for conventional T helper cells (84), suggesting that circulating soluble LAG-3, as well as associated proteins, e.g., STAT4 or IRF4, may be employed as a promising biomarker of MAIT cell activation and disease activity in UC.

In contrast to the proteins that were upregulated in MAIT cell activation, we also identified two protein clusters that were downregulated. These contained mainly proteins connected to the TCR pathway, probably due to ubiquitination processes to down-regulate TCR signalling in the resolution phase of inflammation to avoid chronic activation. Interestingly, we identified C20orf27 (chromosome 20 open reading frame 27), a functionally poorly described protein as central key driver. Recently, C20orf27 has been shown to be involved in cell growth and proliferation of colorectal cancer cells, promoting the activation of the NfKB pathway (85), which is also downstream of the TCR pathway. However, the functional role of this protein in MAIT cells needs further investigations.

Multi-omics data integration has been proposed to reflect a systemic understanding of cellular processes and thus give a more complete view of cellular processes compared to single-omics analyses that reveal only small subsets of effects (86). By integrating multi-omics data, we found a rather moderate positive correlation between transcript and protein expression, with correlation coefficients between 0.2 and 0.4 for all analytes, which is in accordance with other studies (17), suggesting a pivotal role of posttranslational modifications or other regulatory mechanisms, e.g., RNA stability or translational regulation. In this study, the transcriptome of only 5 donors and the proteome of only 4 donors were analyzed. An increased number of donors and transcriptomic and protomics analyses from exactly the same samples may even enhance the certainty of this correlation. However, the here presented correlations between differentially expressed transcripts and proteins were strong (r=0.5-0.7), indicating a consistent regulation of transcription and translation through external stimuli with even small donor numbers. Especially for the Th1 and Th17 pathways, we got a deep coverage of these pathways by multi-omics integration. Notably, we observed the most significant correlation comparing the transcriptome of *E. coli*-activated MAIT cells and the proteome of cytokine-activated MAIT cells, indicating a substantial role of cytokines on the activation and thus, effector functions of MAIT cells in bacterial infections. We, accordingly, suggest considering cytokine effects when studying bacterial activation of MAIT cells *in vitro*.

Furthermore, our data suggest a relevant role of the mode of MAIT cell activation in the pathogenesis of Th1- and Th17-driven diseases. The inflammatory cytokine milieu, e.g., sufficient concentrations of IL-12 and IL-18, at mucosal surfaces where MAIT cells reside, critically shapes T cell activation and differentiation. This is particularly important in diseases, which require MAIT cell-derived IFN-γ for disease control, such as pulmonary infections with *Francisella tularensis* (87) or *Legionella* spp (87, 88), or viral infections as viral hepatitis C (89). In addition, providing sufficient co-stimulatory signals may be a promising strategy to overcome deficient IFN-γ release from circulating MAIT cells as observed in systemic lupus erythematodes (90), inflammatory bowel disease (91), or liver cirrhosis (92).

Certainly, we are aware of some limitations of our study. First, MAIT cells are typically discriminated from other T cell subsets using MR1-5-OP-RU tetramers (8). Here, we identified MAIT cells based on their TCR Vα7.2 and CD161 expression, which might result in minimal cross-contamination with other T cell subsets. Thus, the relevance of presented results, e.g., cytokines or key drivers, should be evaluated in further studies using MR1-5-OP-RU tetramer with larger cohorts *in vitro* and also *in vivo*. Besides, the investigation of different types of infections activating MAIT cells, e.g., viral versus bacterial infections, may be addressed in further studies to evaluate the biological importance, especially of the here identified key drivers.

However, our data set provides a detailed molecular analysis of MAIT cell activation, giving insights into the induction of effector functions and showing that the mode of activation defines a distinct transcriptomic, proteomic and metabolomic profile.

## Data Availability Statement

The datasets presented in this study can be found in online repositories. The names of the repository/repositories and accession number(s) can be found in the article/**Supplementary Material**.

## Ethics Statement

The studies involving human participants were reviewed and approved by Ethics Committee of the University of Leipzig (Ref. #079-15-09032015). The patients/participants provided their written informed consent to participate in this study.

## Author Contributions

KS and MB designed the study, analyzed and interpreted data, and wrote the manuscript. KS, JS, LS, and JH performed experiments for RNA-Seq and data analysis. KS and IK performed proteomics experiments and data evaluation. KS, BE, and UR-K performed metabolomics experiments and data evaluation. IK performed multi-omics integration. TB analyzed and interpreted data. All authors contributed to the article and approved the submitted version.

## Funding

This work was supported by the Helmholtz Association of German Research Centers. MB is grateful for funding by the DFG Collaboration Research Centre SFB 1382/ INST 666/1276 / A05. TB was supported by the German Research Foundation (DFG) (SFB1382 Project ID 403114013/B07).

## Conflict of Interest

The authors declare that the research was conducted in the absence of any commercial or financial relationships that could be construed as a potential conflict of interest.
