# Supplementary material for: A Multi-Omics Analysis of Mucosal-Associated-Invariant T Cells Reveals Key Drivers of Distinct Modes of Activation
Source: Front Immunol. 2021 May 24;12:616967. doi: 10.3389/fimmu.2021.616967 (PMC8183572; doi:10.3389/fimmu.2021.616967)
Supplement: Supplementary file 1 [file DataSheet_1.docx]

Supplementary Material

# Supplementary Data

Supplementary table 1: Transcription factor expression from RNA-Seq data

Supplementary table 2: Cytokine expression from RNA-Seq data

Supplementary table 3: Proteomic data

Supplementary table 4: Metabolomic data

## Supplementary Figures

**SFigure 1: Pathway alterations based on the transcriptome of activated MAIT cells.** Hierarchical clustering and heatmap of enriched core pathways of DE genes during MAIT cell activation determined by IPA (p.adj ≤0.05, z-scores).

**SFigure 2: Expression levels of detected cytokines and chemokines.** Differentially expressed (p.adj ≤0.01) cytokines and chemokines in a-CD3/a-CD28- or *E.coli*-activated MAIT cells were ranked as low, medium and highly expressed based on an empirically determined threshold of raw counts: low ≤ 4K raw counts, medium >4K - <10k raw counts, high ≥10k raw counts, applied to the mean of the raw counts of samples belonging to the same treatment. Depicted are the normalized raw counts (rlogs) of the three treatments.

SFigure 3: Weighted Gene Correlation Network Analysis (WGCNA) of the transcriptome of activated MAIT cells. (a) Consensus dendrogram showing the gene tree with corresponding color-coded modules. (b) Module eigengene clustering showing the merged modules for a-CD3/a-CD28 in blue and for *E.coli* in red. (c) Gene counts of merged modules for a-CD3/a-CD28 and for *E.coli* from WGCNA. (d) The module membership (MM) of the a-CD3/a-CD28 and the *E.coli* module were plotted against the gene significance (GS). Putative key drivers (hub genes) were considered as the 50 genes with the highest absolute GS and MM values.

SFigure 4: Weighted Gene Correlation Network Analysis (WGCNA) of the proteome of activated MAIT cells. (a) Consensus dendrogram showing the protein tree with corresponding modules that were assigned to colors. (b) Protein counts of modules from WGCNA. (c) The module membership (MM) of the turquoise and the blue module was plotted against the protein significance (PS) of the unstimulated control (ctrl) or IL-12/IL-18 + a-CD3/a-CD28-activated MAIT cell samples. Putative key drivers (hub proteins) were considered with absolute PS and MM values ≥0.5. (d) Log2-transformed LFQ intensity of selected key drivers (mean +SD, n = 5). Level of significances are given as p-values with ****≤0.0001, ***≤0.001, **≤0.01, *≤0.05.

SFigure 5: Correlation of the Th1 and Th17 activation pathway between transcriptome and proteome of activated MAIT cells. Proteins and genes that were assigned to the pathway in IPA and either differential in proteome or transcriptome were used for the correlation.

**SFigure 6**: **Metabolomic profiling of MAIT cell activation.** (a) Principal component analysis of the metabolome of MAIT cells stimulated with IL-12/IL-18, a-CD3/a-CD28 or a combination of both. Each point represents a single donor (n=4). Overlap of (b) all identified metabolites and (c) differentially abundant metabolites in stimulated MAIT cells. (d) Volcano plot highlighting differentially abundant metabolites in stimulated MAIT cells (p-value ≤0.05).
