# Supplementary figures and images for: A Multi-Omics Analysis of Mucosal-Associated-Invariant T Cells Reveals Key Drivers of Distinct Modes of Activation
Source: Front Immunol. 2021 May 24;12:616967. doi: 10.3389/fimmu.2021.616967 (PMC8183572; doi:10.3389/fimmu.2021.616967)

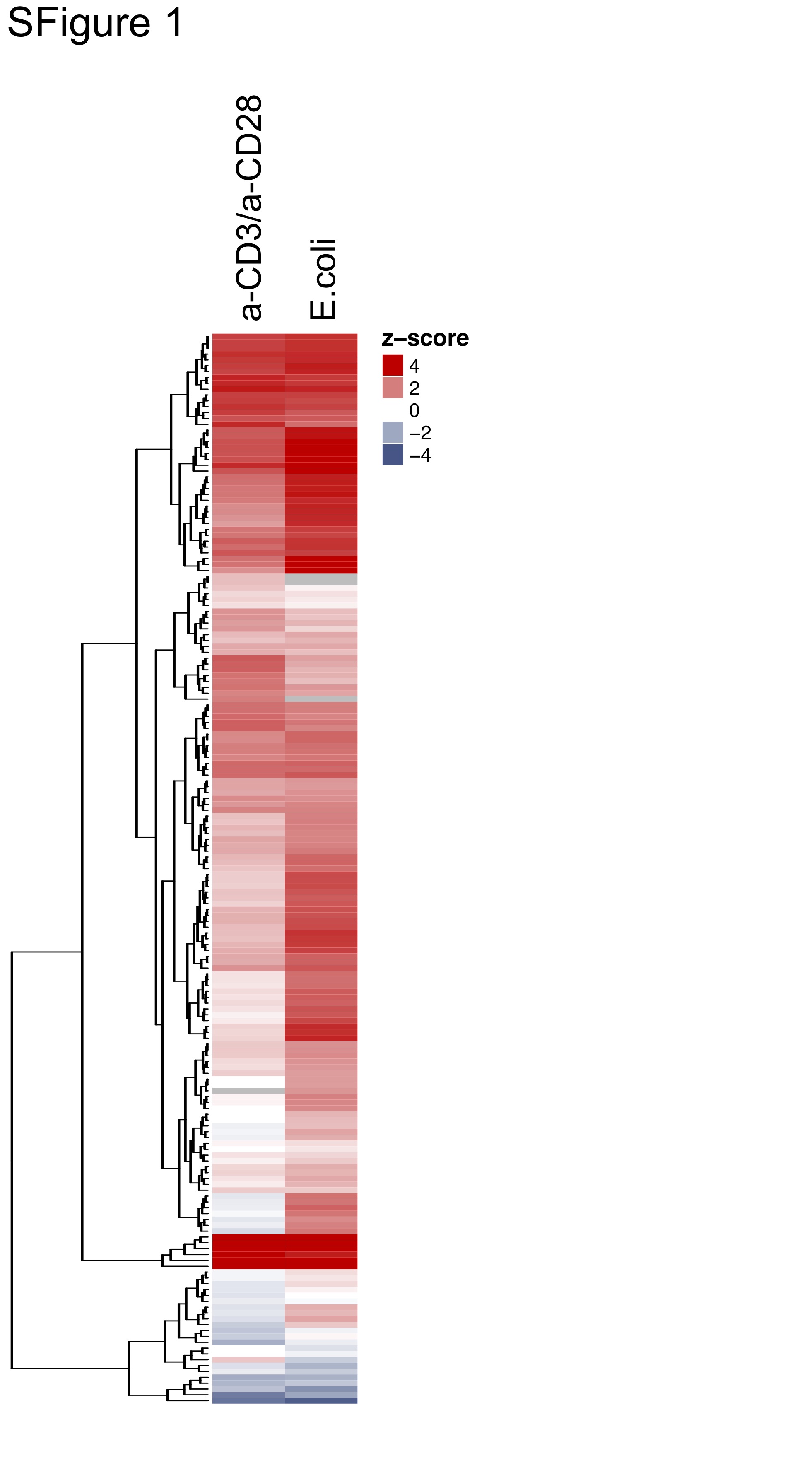

Supplement: Supplementary file 2 [file Image_1.jpeg]

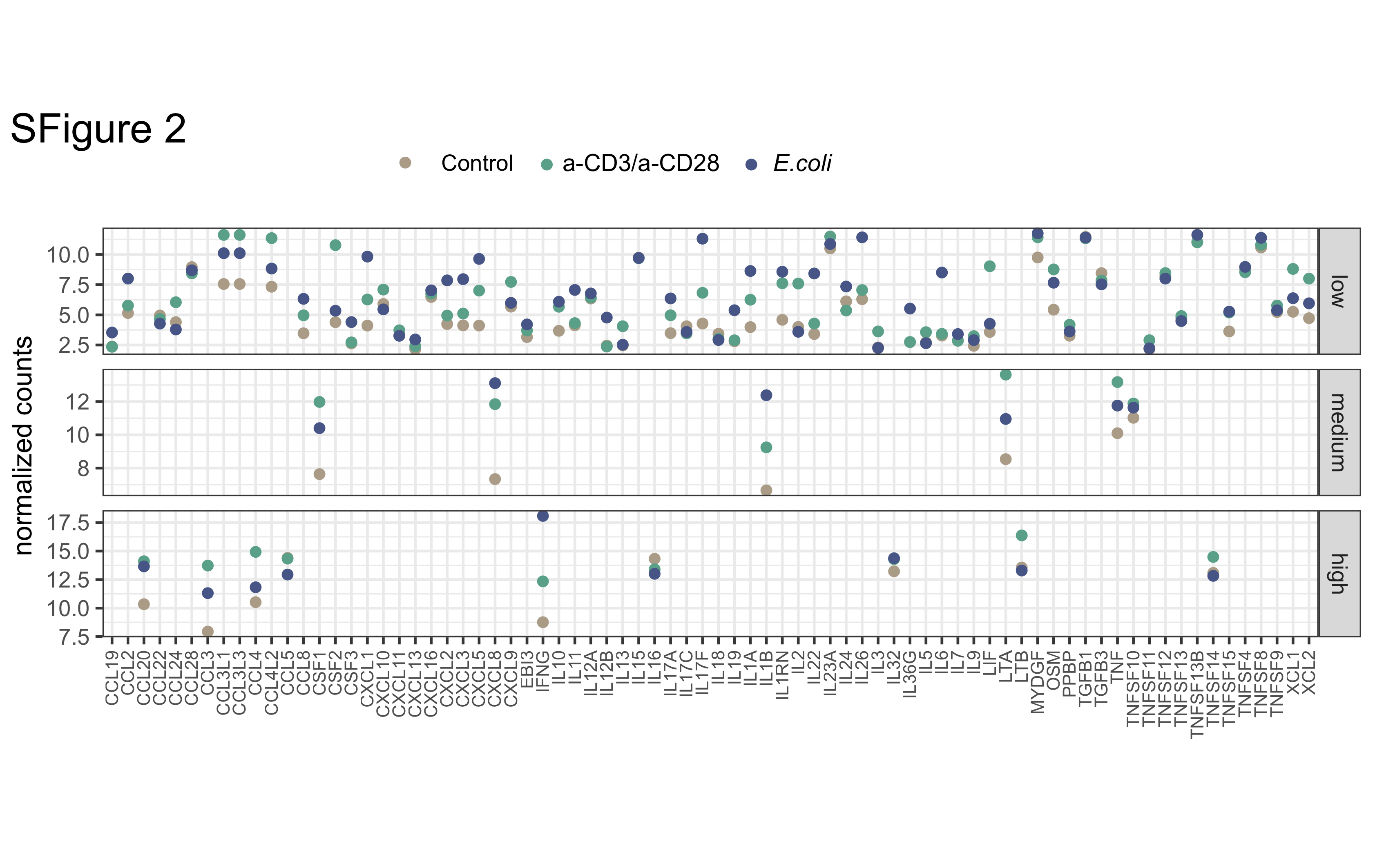

Supplement: Supplementary file 3 [file Image_2.jpeg]

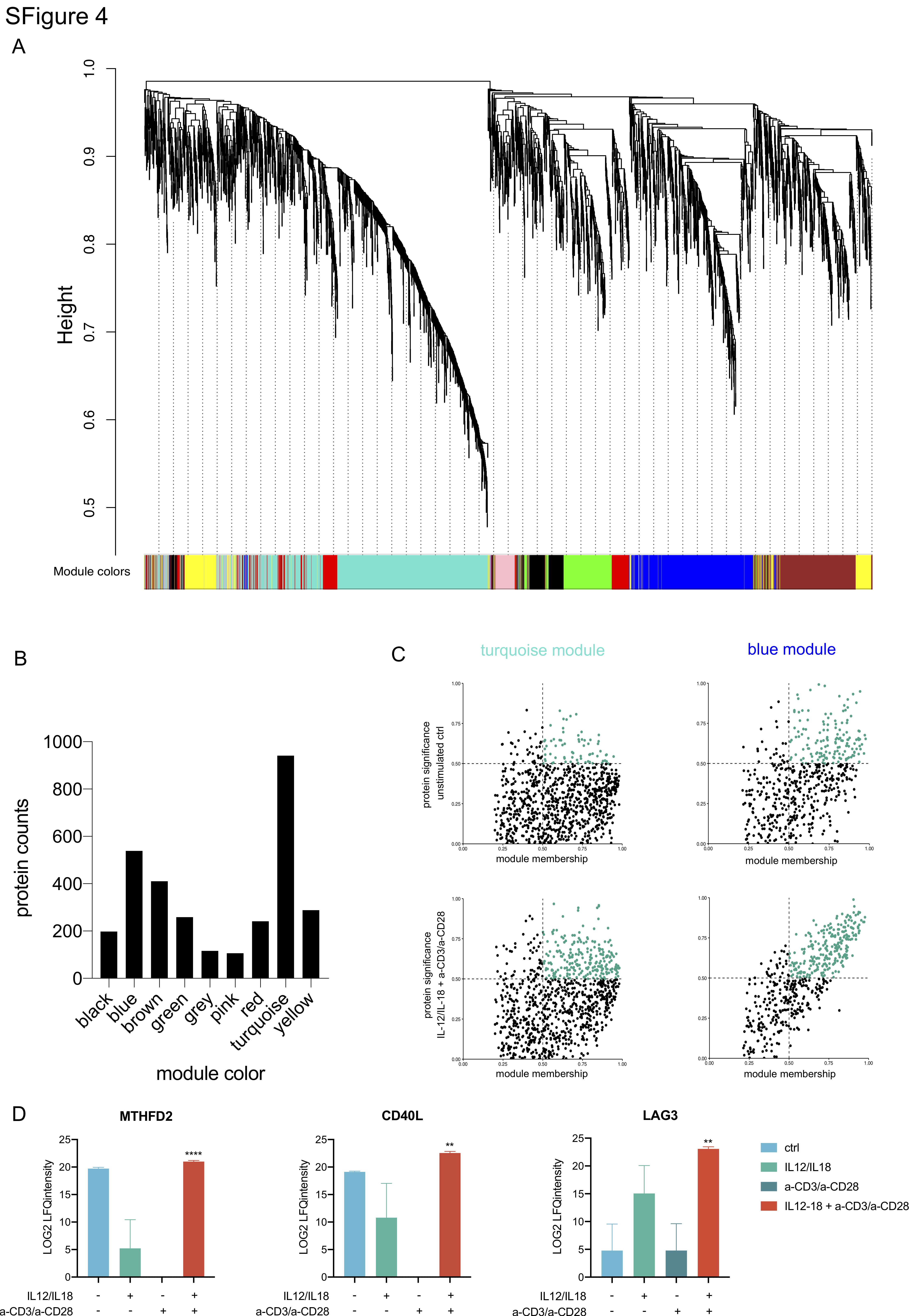

Supplement: Supplementary file 5 [file Image_4.jpeg]

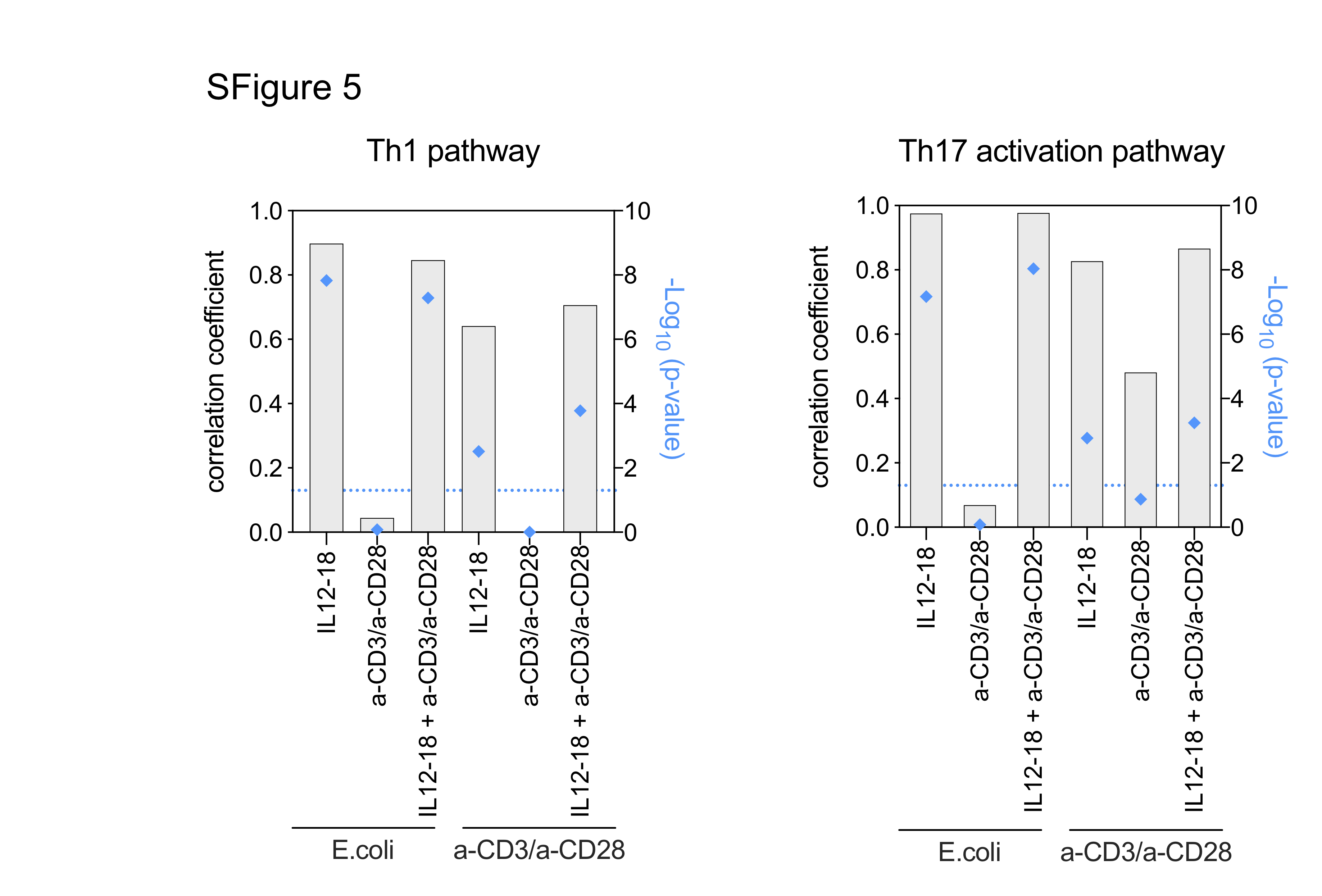

Supplement: Supplementary file 6 [file Image_5.jpeg]

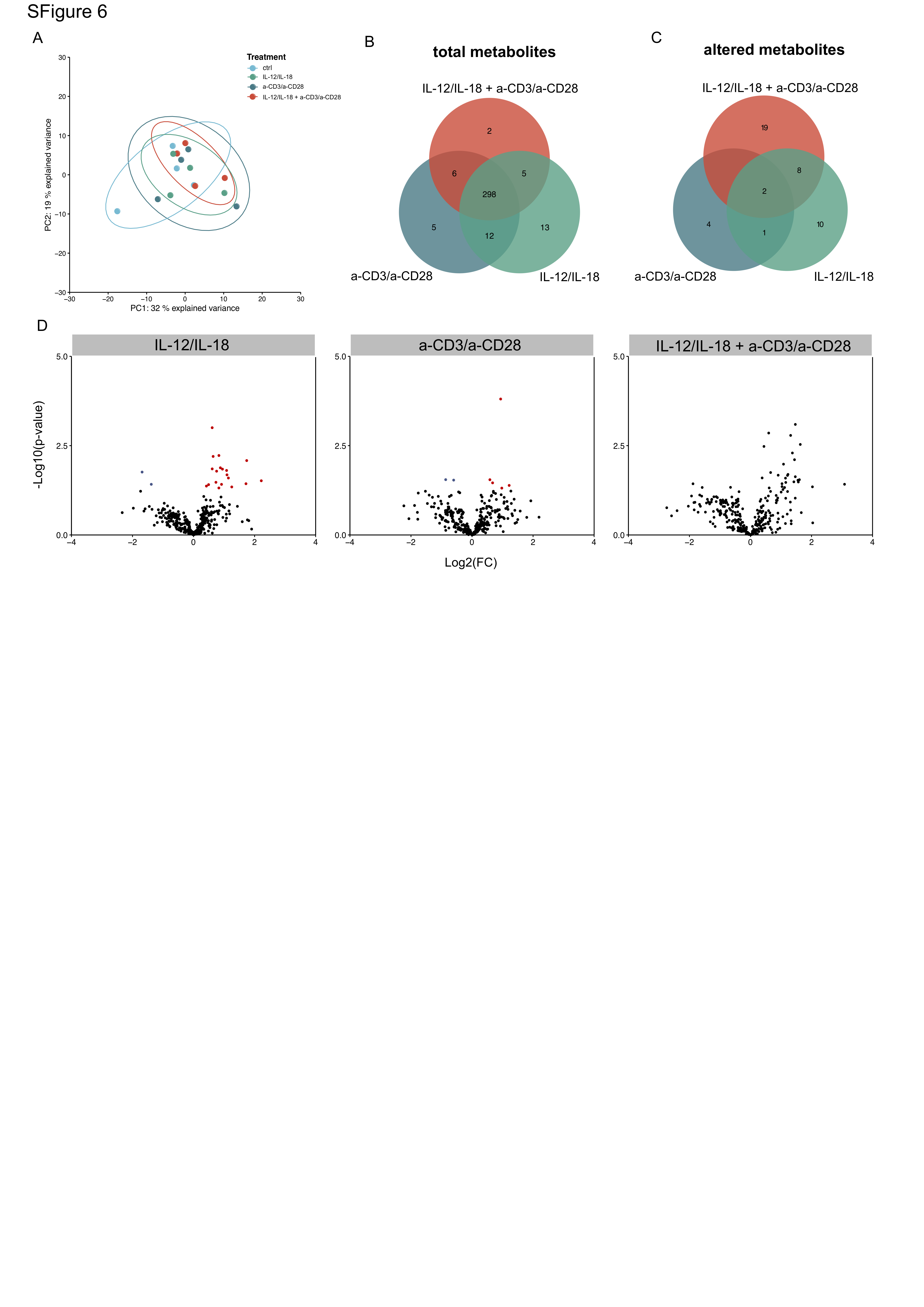

Supplement: Supplementary file 7 [file Image_6.jpeg]
